# Supplementary material for: Small-molecule-biased formyl peptide receptor agonist compound 17b protects against myocardial ischaemia-reperfusion injury in mice
Source: Nat Commun. 2017 Feb 7;8:14232. doi: 10.1038/ncomms14232 (PMC5309721; doi:10.1038/ncomms14232)
Supplement: Supplementary Information — Supplementary Tables and Supplementary Figures. [file ncomms14232-s1.pdf]

SUPPLEMENTARY RESULTS

Supplementary Table 1.

	hFPR1- FlpIn-CHO					hFPR2-FlpIn-CHO				
	**pEC_{50}**	**E_{max} (%)**	**$Log(\tau/K_A)$**	**$\Delta Log(\tau/K_A)$**	**N**	**pEC_{50}**	**E_{max} (%)**	**$Log(\tau/K_A)$**	**$\Delta Log(\tau/K_A)$**	**n**
**ERK1/2 phosphorylation**										
fMLP	9.0±0.6	80±7	9.6±0.2	2.3±0.1	6	5.2±0.1	68±11	5.4±0.1	-2.3±0.2	4
Cmpd17b	6.1±0.2**	82±6	6.0±0.2	-1.3±0.2^^^	6	5.4±0.4	66±18	5.8±0.1	-1.9±0.3^^	4
Cmpd43	7.2±0.2***	61±9	7.3±0.1	0.0	6	7.3±0.4**#	95±4	7.7±0.2	0.0	4
**Akt 1/2/3 (Thr308) phosphorylation**										
fMLP	8.9±0.3	18±4	8.8±0.3	1.9±0.1	5	4.7±0.2	21±2	4.8±0.1	-2.1±0.3	5
Cmpd17b	5.7±0.2****	24±6	6.0±0.1	-0.9±0.2^^	5	4.8±0.1	21±3	5.1±0.2	-1.9±0.2^^	5
Cmpd43	6.9±0.3**#	15±4	6.9±0.3	0.0	5	6.9±0.2**#	27±2	7.0±0.2	0.0	5
**Akt 1/2/3 (Ser473) phosphorylation**										
fMLP	9.0±0.5	16±4	9.0±0.4	2.1±0.3	4	5.0±0.1	20±4	4.9±0.2	-2.0±0.2	5
Cmpd17b	5.8±0.2****	18±7	5.7±0.2	-1.3±0.3^^	5	5.0±0.1	20±3	5.1±0.2	-1.9±0.2^^^	5
Cmpd43	7.1±0.3**#	13±3	6.9±0.3	0.0	5	6.8±0.3**#	30±6	6.9±0.4	0.0	5
**$[Ca^{2+}]_i$ mobilization**										
fMLP	9.0±0.2	38±6	9.4±0.3	1.5±0.7	4	5.8±0.1	38±4	5.4±0.1	-2.2±0.3	3
Cmpd17b	5.8±0.2****	31±4	5.5±0.2	-2.5±0.1	7	5.2±0.1	16±2***	4.3±0.1	-3.4±0.3	4
Cmpd43	8.0±0.2**####	43±6	8.0±0.2	0.0	7	7.7±0.3**####	51±3*#####	7. 7±0.3	0.0	5
**Inhibition of cAMP accumulation**										
fMLP	7.3±0.0	28±12	8.6±0.3	1.5±0.5	3	5.5±0.5	10±8	4.9±0.1	-1.6±0.2	4
Cmpd17b	6.3±0.2	12±10	5.8±0.4	-1.3±0.6^^	4	4.9±0.1	47±6**	5.1±0.1	-1.4±0.2^^^	4
Cmpd43	7.1±0.4	27±20	7.1±0.3	0.0	3	6.8±0.1*##	66±6***	6.5±0.1	0.0	4

FPR agonist potency (pEC_{50}), maximal agonist response (E_{max}), transduction coefficient ($Log(\tau/K_A)$), and "normalized" transduction coefficient ($\Delta Log(\tau/K_A)$) for phosphorylation of ERK1/2, Akt1/2/3(Thr308), Akt1/2/3(Ser473), intracellular Ca^{2+} mobilization and inhibition of cAMP

accumulation in CHO cells stably expressing hFPR1 or hFPR2. Data represents percent of the mean±SEM of response elicited by 10% FBS (phosphorylation), ATP (Ca^{2+} mobilization) or inhibition of forskolin-induced cAMP accumulation, from 3-7 experiments performed in triplicate, *n* indicates number of independent experiments. **p<0.01, ****p<0.0001 vs. fMLP; $^{###}$p<0.001, $^{####}$p<0.0001 vs Cmpd17b, ^^p<0.01, ^^^p<0.001 vs Δ Log(τ/K_A) estimated for the corresponding agonist at intracellular Ca^{2+} mobilization, one-way ANOVA followed by Tukey's *post-hoc* test.

Supplementary Table 2.

		Sham	I-R	I-R+ Cmpd17b	I-R+Cmpd43
48 h myocardial I-R					
n		6	9	10	7
Body Weight (BW, g)		27±1	29±1	28±1	27±1
Organ weight:BW (mg per g)	heart	4.2±0.2	4.6±0.2$^{\#}$	5.2±0.3$^{\#}$	4.8±0.3
	atria	0.3±0.0	0.4±0.0	0.5±0.0	0.5±0.1
	LV	3.1±0.1	3.6±0.1	3.7±0.2	3.5±0.2
	RV	0.8±0.1	0.8±0.1	1.0±0.0	0.8±0.0
	lung	5.2±0.2	5.4±0.1	5.6±0.2	5.7±0.1
7 d myocardial I-R					
n		6	14	8	8
BW (g)		26±1	26±1	24±1	24±1
Organ weight:BW (mg per g)	heart	4.6±0.2	5.2±0.2	4.0±0.1*	5.0±0.2
	Atria	0.2±0.0	0.4±0.0$^{\#}$	0.3±0.0	0.3±0.0
	LV	3.3±0.2	3.9±0.2	2.9±0.1*	3.7±0.1
	RV	0.8±0.1	0.9±0.1	0.8±0.3	0.9±0.1
	lung	5.8±0.3	5.6±0.2	5.4±0.1	5.8±0.3

Impact of I-R in the presence and absence of Cmpd17b and Cmpd43 treatment on body and organ weights 48h and 7 days following I-R injury. $^{\#}$P<0.05 vs sham and *P<0.05 vs vehicle-treated I-R mice. One-way ANOVA with Tukey's *post-hoc* test. Data were represented as mean±SEM, *n* indicates number of mice.

Supplementary Table 3.

		Sham	MI	MI+Cmpd17b
n		10	13	13
Body Weight (BW, g)		29.7±0.8	27.3±0.5$^{\#}$	28.0±0.7
Organ weight:BW (mg per g)	heart	4.5±0.1	5.9±0.3$^{\#\#\#\#}$	5.4±0.1$^{\#\#}$
	atria	0.3±0.0	0.5±0.0$^{\#\#\#\#}$	0.4±0.0$^{\#\#}$
	LV	3.3±0.1	4.4±0.1$^{\#\#\#\#}$	4.0±0.1$^{\#\#\#}$
	RV	0.8±0.1	0.9±0.0	0.9±0.0
	lung	3.3±0.1	4.4±0.1$^{\#\#\#\#}$	4.0±0.1$^{\#\#\#*}$
Echocardiographic analysis				
		Sham	MI	MI+Cmpd17b
HR (beats per min)		616±14	568±19	598±14
LVEDD (mm)		3.9±0.1	4.5±0.1$^{\#}$	4.6±0.1$^{\#}$
LVESD (mm)		2.2±0.2	3.3±0.1$^{\#\#\#}$	3.0±0.2$^{\#\#}$
LVPW (mm)		1.3±0.1	1.2±0.1	1.0±0.0*

Impact of MI in the presence and absence of Cmpd17b on endpoint body and organ weights, as well as echocardiographic parameters of LV function in anesthetized mice at study endpoint, 4 weeks following permanent LAD occlusion. LVEDD, LV end-diastolic dimension; LVESD, LV end-systolic dimension; LVPW, LV posterior wall; $^{\#}$P<0.05 vs sham and *P<0.05 vs vehicle-treated MI mice. One-way ANOVA with Tukey's *post-hoc* test. Data were represented as mean±SEM, *n* indicates number of mice.

Supplementary Table 4.

	**Forward primer**	**Reverse primer**
**18S**	TGTTCACCATGAGGCTGAGATC	TGG TTG CCT GGG AAA ATC C
**mβ-MHC**	TCT CCT GCT GTT TCC TTA CTT GCT A	GTA CTC CTC TGC TGA GGC TTC CT
**mCTGF**	TGA CCC CTG CGA CCC ACA	TAC ACC GAC CCA CCG AAG ACA CAG
**mTGFβ**	TGGAGCAACATGTGGAACTC	GTCAGCAGCCGGTTACCA
**mIL-1β**	TTGACGGACCCCAAAAGAT	GAAGCTGGATGCTCTCATCCTG
**mFPR1**	CCTTGGCTTTCTTCAACAGC	GCCCGTTCTTTACATTGC AT
**mFPR2**	ACA GCA GTT GTG GCT TCCTT	CCT GGC CCA TGA AAA CAT AG
**mCD68**	CCAATTCAGGGTGGAAGAAA	CTCGGGCTCTGATGTAGGTC
**mTNFα**	CTG TAG CCC ACG TCG TAG C	TTG AGA TCC ATG CCG TTG

5’-3’ primer sequences used for gene expression by qrt-PCR.

Supplementary Table 5.

	**Gateway entry vector 5'**	**Stop in gateway entry vector 3'**
**FPR1**	ggggacaagtttgtacaaaaaagcaggcttcCACCATGGAGACAAATTCCTCTCTCCCCACG	ggggaccactttgtacaagaaagctgggtcTCACTTTGCCTGTAACTCCACCTCTGC
**FPR2**	ggggacaagtttgtacaaaaaagcaggcttcCACCATGGAAACCAACTTCTCCACTCC	ggggaccactttgtacaagaaagctgggtcTCACATTGCCTGTAACTCAGTCTCTGC
**FPR3**	ggggacaagtttgtacaaaaaagcaggcttcCACCATGGAAACCAACTTCTCCATTC	ggggaccactttgtacaagaaagctgggtcTCACATTGCTTGTAACTCCGTCTCCTC

Gateway entry vector used for generating the hFPR cell line. The capital letters reflect the FPR sequence whereas the small letters are the gateway entry vector sequence required to insert the gene into the gateway entry vector plasmid.

Supplementary Figures

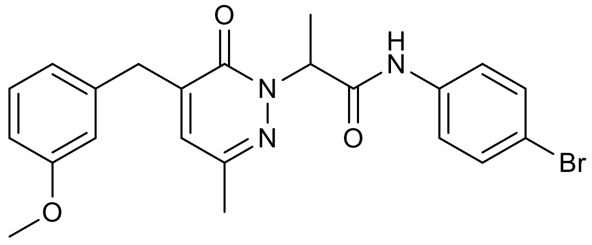

Cmpd17b
Molecular weight: 456.34

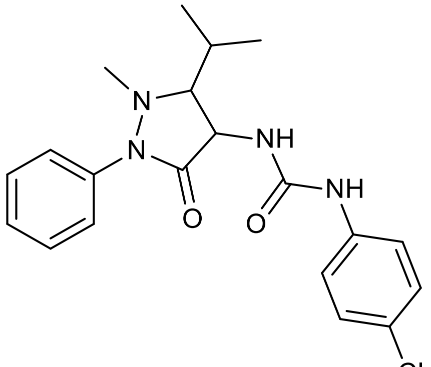

Cmpd43
Molecular weight: 384.87

Supplementary Figure 1. Chemical structures of small molecule FPR agonists *N*-(4-bromophenyl)-2-[5-(3-methoxybenzyl)-3-methyl-6-oxo-6H-pyridazin-1-yl]-propionamide, identified as compound-17b (Cmpd17b) and the Amgen pyrazolone derivative, identified as compound-43 (Cmpd43).

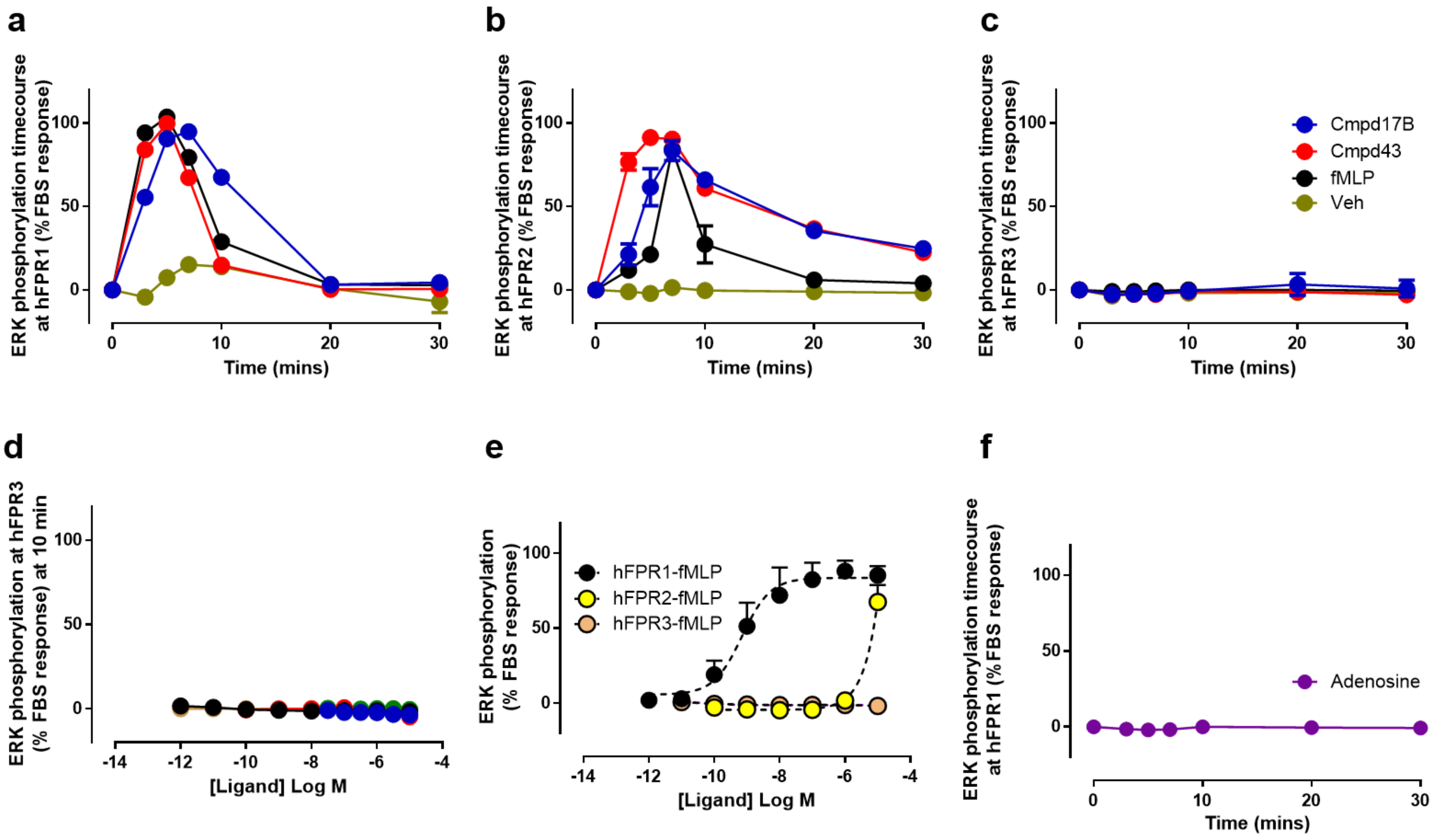

a
ERK phosphorylation timecourse at hFPR1 (% FBS response)
100
50
0
0
10
20
30
Time (mins)
b
ERK phosphorylation timecourse at hFPR2 (% FBS response)
Time (mins)
c
ERK phosphorylation timecourse at hFPR3 (% FBS response)
Cmpd17B
Cmpd43
fMLP
Veh
Time (mins)
d
ERK phosphorylation at hFPR3 (% FBS response) at 10 min
-14
-12
-10
-8
-6
-4
[Ligand] Log M
e
ERK phosphorylation (% FBS response)
hFPR1-fMLP
hFPR2-fMLP
hFPR3-fMLP
[Ligand] Log M
f
ERK phosphorylation timecourse at hFPR1 (% FBS response)
Adenosine
Time (mins)

Supplementary Figure 2. ERK1/2 phosphorylation in hFPR-CHO cells. Time-course of ERK1/2 phosphorylation in response to fMLP, Cmpd17b, and Cmpd43 (each 10 µM, n=4 separate experiments) in **a** hFPR1-CHO, **b** hFPR2-CHO and **c** FPR3-CHO cells. **d** Concentration-response curves for each FPR agonist in hFPR3-CHO cells (results expressed as mean ± S.E.M. of 3-4 experiments each performed in triplicate). For panels a-d, fMLP (black symbols); Cmpd17b (blue symbols); Cmpd43 (red symbols); 10%DMSO vehicle (brown symbols). **e** Concentration-response curves to fMLP (dotted lines; hFPR1, hFPR2 and hFPR3 in black, yellow and beige respectively) on ERK1/2 phosphorylation in hFPR subtypes. **f** Concentration-response curves to the GPCR agonist adenosine in hFPR1-CHO cells on ERK1/2 phosphorylation (results expressed as mean ± S.E.M. of 3 experiments each performed in triplicate).

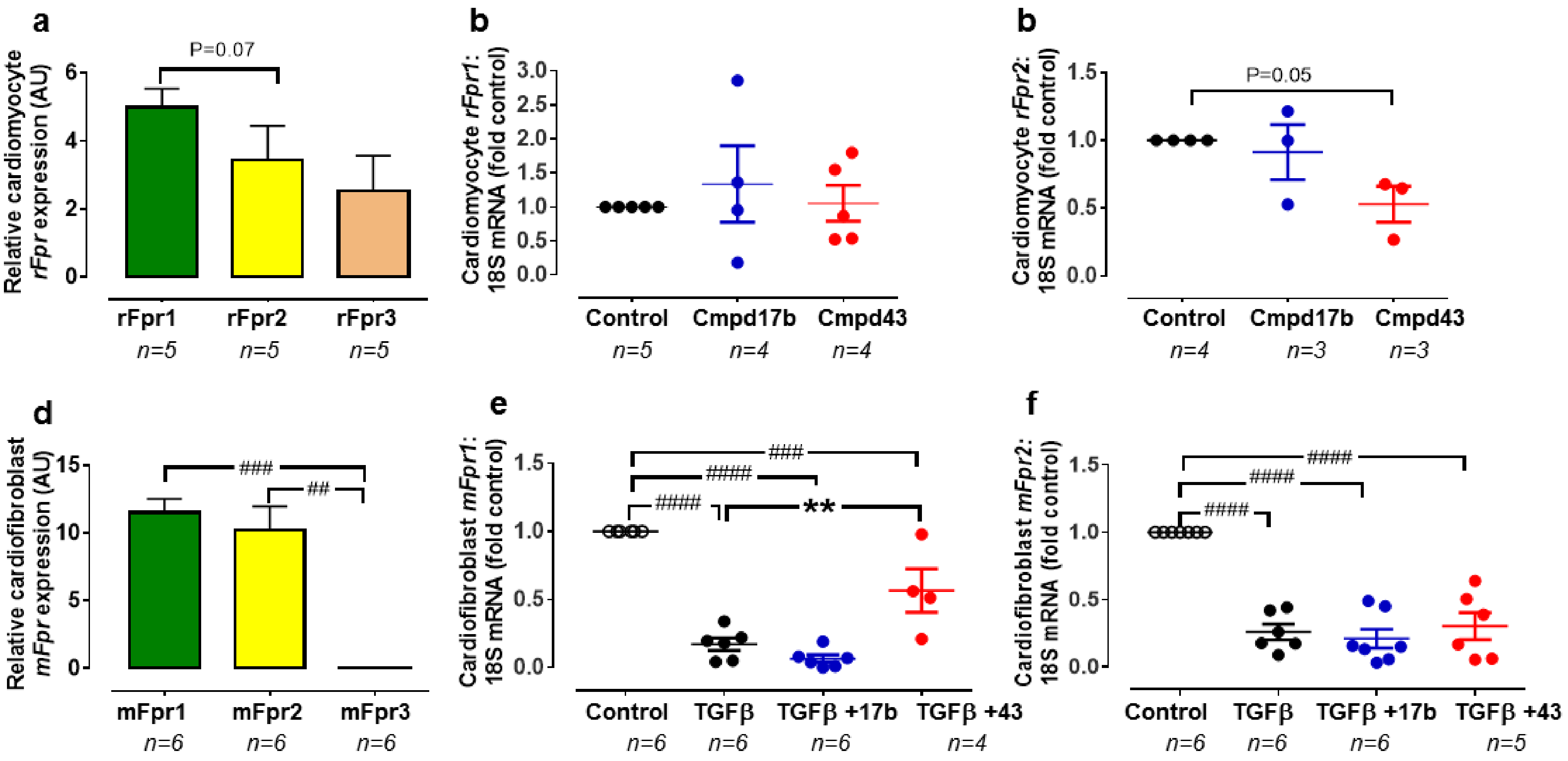

a
Relative cardiomyocyte rFpr expression (AU)
P=0.07
rFpr1
rFpr2
rFpr3
n=5
n=5
n=5
b
Cardiomyocyte rFpr1: 18S mRNA (fold control)
Control
Cmpd17b
Cmpd43
n=5
n=4
n=4
b
Cardiomyocyte rFpr2: 18S mRNA (fold control)
P=0.05
Control
Cmpd17b
Cmpd43
n=4
n=3
n=3
d
Relative cardiofibroblast mFpr expression (AU)
###
##
mFpr1
mFpr2
mFpr3
n=6
n=6
n=6
e
Cardiofibroblast mFpr1: 18S mRNA (fold control)
###
####
####
**
Control
TGFβ
TGFβ +17b
TGFβ +43
n=6
n=6
n=6
n=4
f
Cardiofibroblast mFpr2: 18S mRNA (fold control)
####
####
####
Control
TGFβ
TGFβ +17b
TGFβ +43
n=6
n=6
n=6
n=5

Supplementary Figure 3. Cardiomyocyte expression of Fprs *in vitro.* a Relative expression of rat *Fpr*s (*rFpr1, rFpr2* and *rFpr3* shown in green, yellow and beige bars, respectively), in untreated NRCM determined via real-time PCR (expressed as the threshold cycle number Ct subtracted from the maximum cycle number utilized, 40). Impact of 48 h incubation with Cmpd17b (blue symbols, 1 μM) and Cmpd43 (red symbols, 1 μM) on NRCM expression of **b** *rFpr1* and **c** *rFpr2*, expressed as fold change vs vehicle-treated cells (black symbols). **d** Relative expression of mouse *Fpr*s (*mFpr1,mFpr2 and mFpr3,* shown in green, yellow and beige bars, respectively) in AMCF determined via real-time PCR. Impact of 24 h incubation with TGF-β (black symbols) ± Cmpd17b (blue symbols, 10 μM) and Cmpd43 (red symbols, 10 μM) on AMCF expression of **e** *mFpr1* and **F** *mFpr2*, fold vehicle-treated cells (open symbols). $^{\#}$P<0.05, $^{\#\#}$P<0.01, $^{\#\#\#}$P<0.001 and $^{\#\#\#\#}$P<0.0001 vs vehicle, *P<0.05 vs TGF-β, One-way ANOVA with Tukey's *post-hoc* test. Data represented as mean ± S.E.M. (n per number of separate experiments indicated below x-axis).

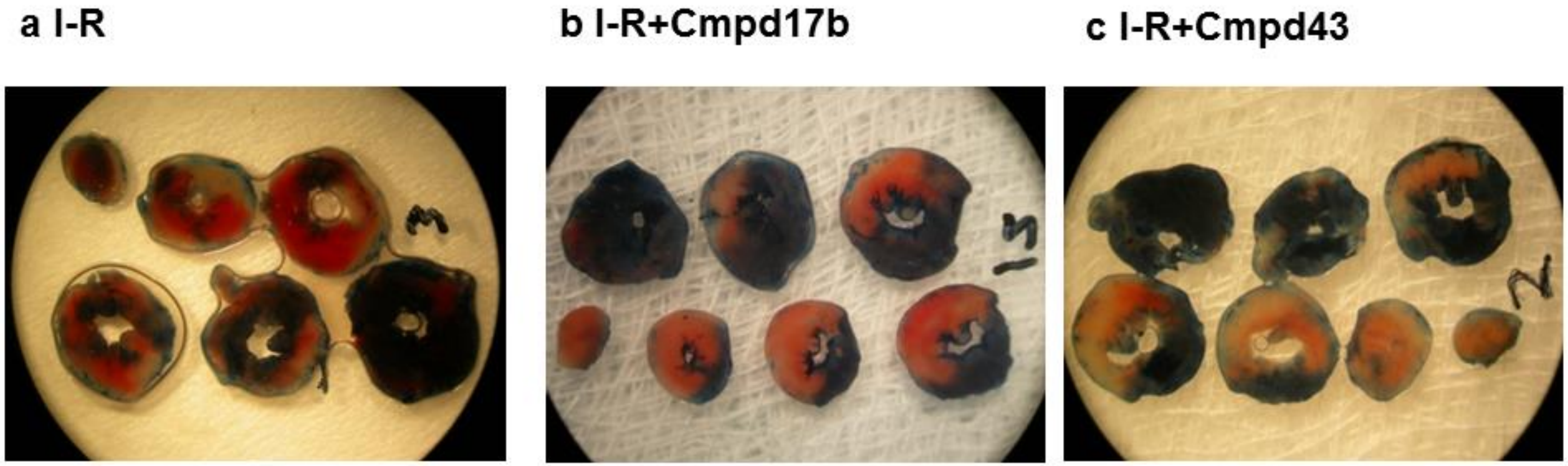

Supplementary Figure 4. Impact of FPR agonist Cmpd17b on cardiac necrosis 24 h after I-R *in vivo*. Transverse LV slices obtained from one representative mouse per group, subjected to 40 min ischemia followed by 24 h reperfusion, in the vehicle, Cmpd17b or Cmpd43 (both 50 mg per kg i.p.) treatment groups. Three different zones were visible after staining with Evans blue and TTC. The areas stained dark blue, white and red represented non-risk zones, infarct zones and ischemic but non-infarcted zones, respectively. The risk zone was calculated as a percentage of red plus the white area over the total LV.

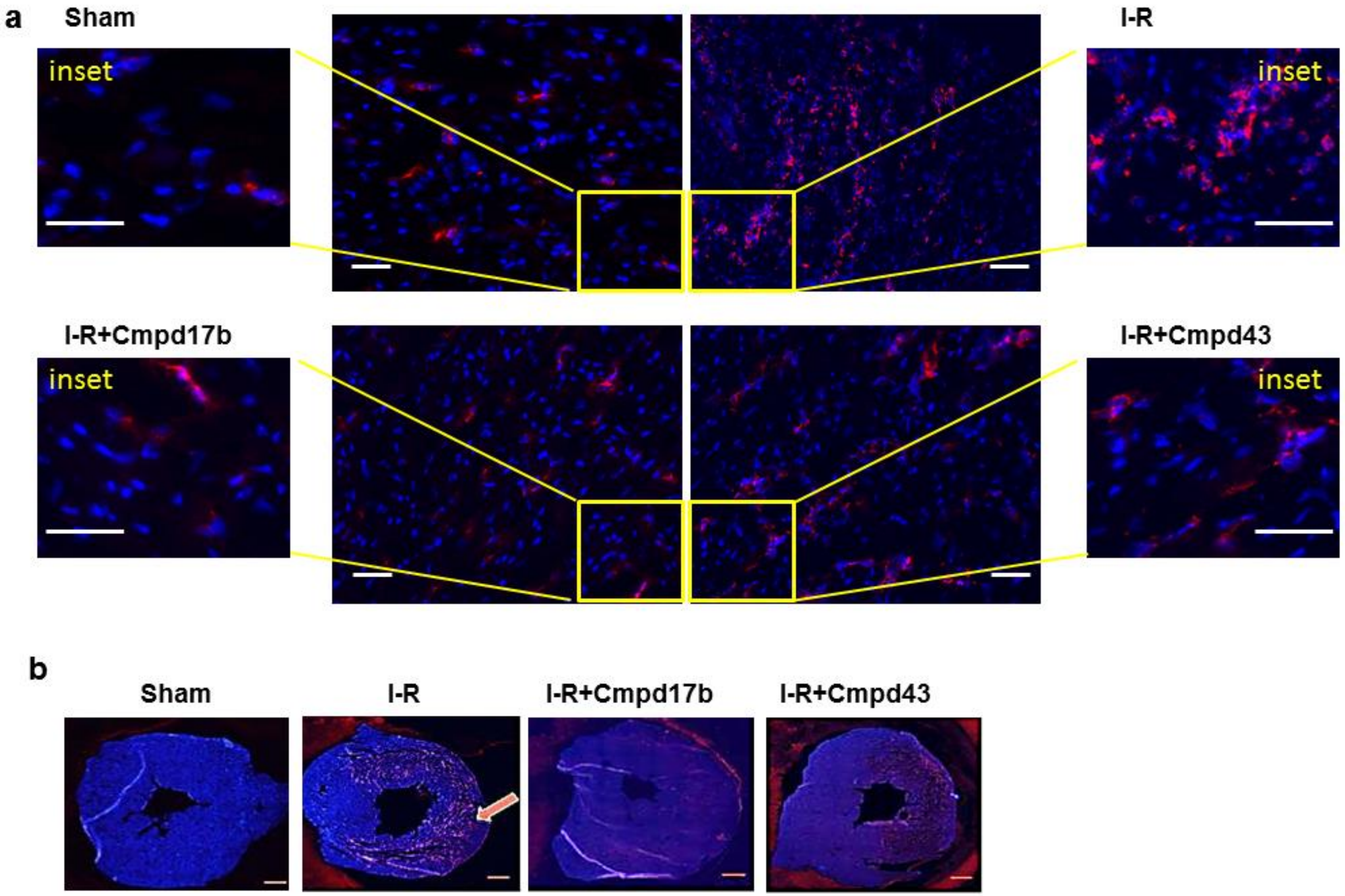
a
Sham
inset
I-R
inset
I-R+Cmpd17b
inset
I-R+Cmpd43
inset
b
Sham
I-R
I-R+Cmpd17b
I-R+Cmpd43

Supplementary Figure 5. Impact of FPR agonist Cmpd17b on cardiac neutrophil content 48 h after I-R *in vivo*. a Representative immunofluorescent images of LV neutrophil content, from sham, vehicle, and FPR agonist treated (Cmpd17b or Cmpd43, both 50 mg per kg per day, i.p.) mice, 48 h post I-R, detected using an anti-Ly6B.2 antibody at 40x magnification; inset highlights the area at risk (one representative mouse per group, scale bars: 100 µm). **b** Representative immunofluorescent images of LV neutrophil content 48 h post I-R, depicting stitched 9x9 single images under 20x magnification (one representative mouse per group, scale bars: 500 µm). Arrow indicates infarct region.

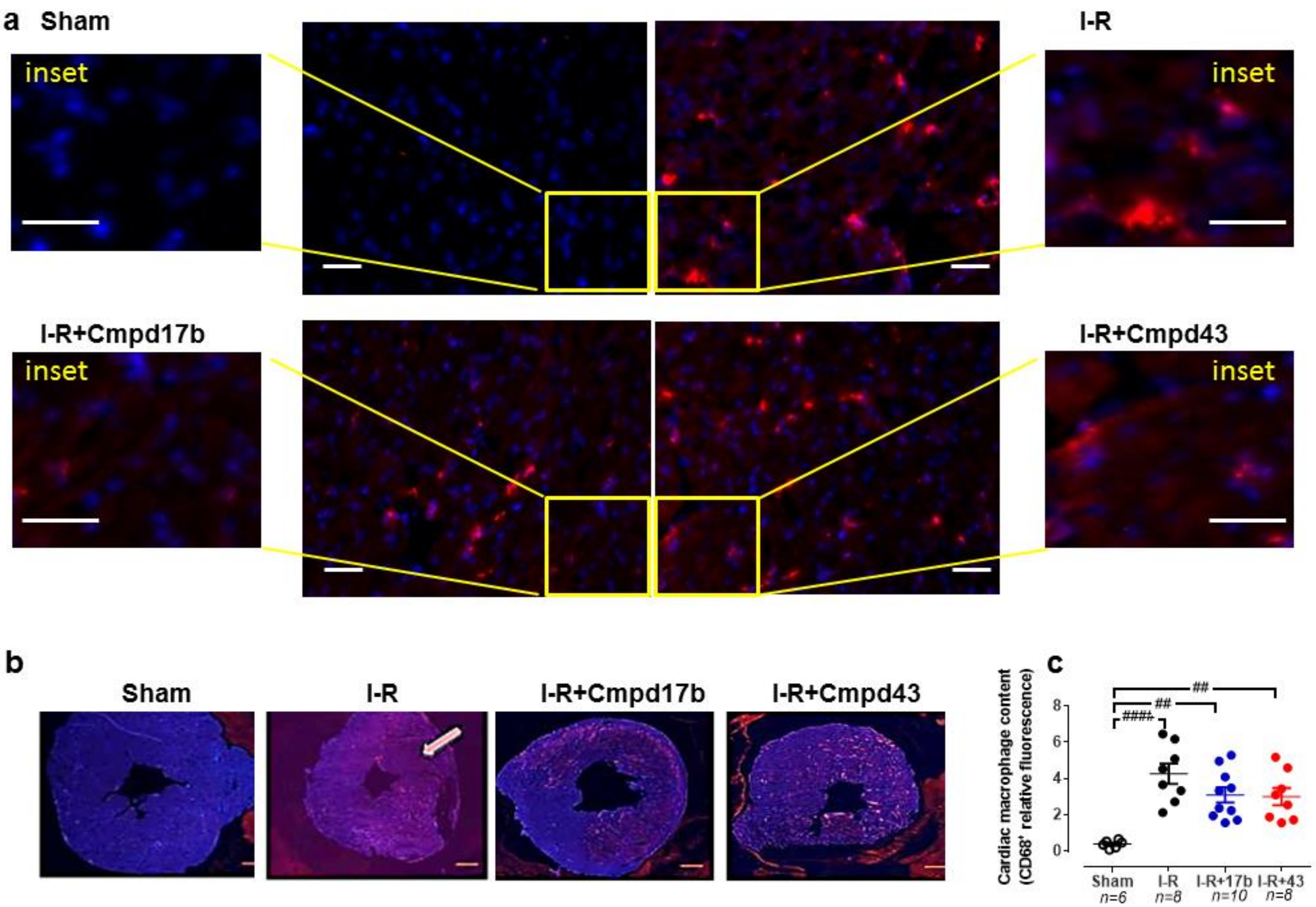
a
Sham
inset
I-R
inset
I-R+Cmpd17b
inset
I-R+Cmpd43
inset
b
Sham
I-R
I-R+Cmpd17b
I-R+Cmpd43
c
Cardiac macrophage content
(CD68+ relative fluorescence)
##
##
####
8
6
4
2
0
Sham
n=6
I-R
n=8
I-R+17b
n=10
I-R+43
n=8

Supplementary Figure 6. Impact of FPR agonist Cmpd17b on cardiac macrophage content 48 h after I-R *in vivo*. a Representative immunofluorescent images of LV macrophage content, from sham, vehicle, and FPR agonist treated (Cmpd17b or Cmpd43, both 50 mg per kg per day, i.p.) mice, 48 h post I-R, detected using an anti-CD68+ antibody at 40x magnification; inset highlights the area at risk (one representative mouse per group, scale bars: 100 µm). **b** Representative immunofluorescent images of LV macrophage content 48h post I-R, depicting stitched 9x9 single images under 20x magnification (scale bars: 500 µm; arrow indicates infarct region, one representative mouse per group). **c** Pooled data for LV CD68-positive immunofluorescence (determined from images under 40x magnification), from sham (open symbols), vehicle- (black symbols), and FPR agonist-treated (Cmpd17b or Cmpd43, both 50 mg per kg per day, i.p.; blue and red symbols, respectively) mice, 48 h post I-R. Results are expressed as mean ± S.E.M., with n per group indicated. $^{##}P<0.01$ and $^{####}P<0.0001$ vs sham mice on one-way ANOVA with Tukey's *post-hoc* test.

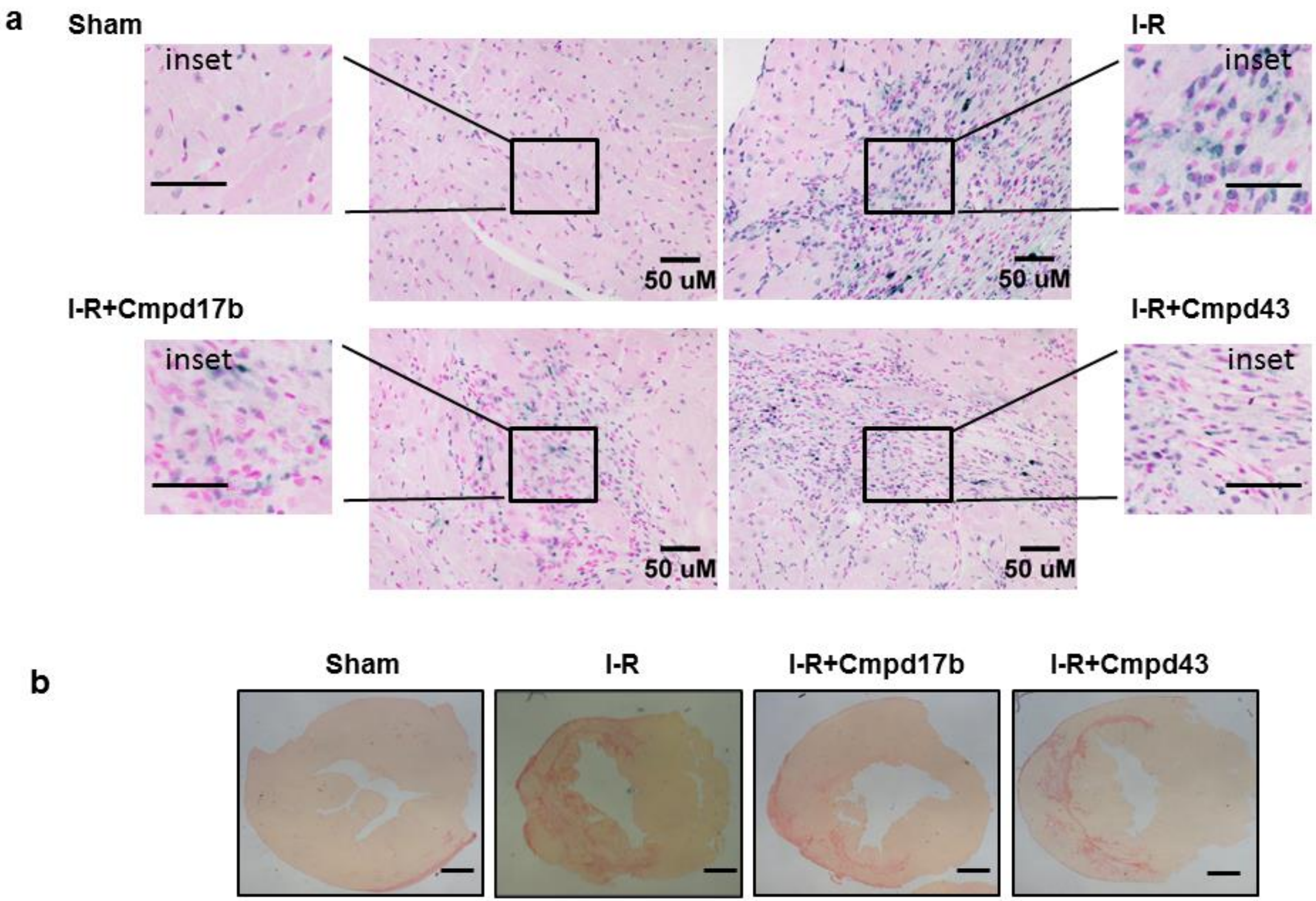
a
Sham
inset
50 uM
I-R
inset
50 uM
I-R+Cmpd17b
inset
50 uM
I-R+Cmpd43
inset
50 uM
b
Sham
I-R
I-R+Cmpd17b
I-R+Cmpd43

Supplementary Figure 7. Impact of FPR agonist Cmpd17b on cardiac injury 7-days after I-R *in vivo*. a Higher magnification (200x) representative CardioTAC-stained LV sections showing dead:viable cells from sham, vehicle- and FPR agonist (Cmpd17b or Cmpd43, both 50 mg per kg per day i.p.)-treated mice, 7 d post I-R (one representative mouse per group; scale bars: 100 µm). Inset highlights a region within the area at risk. **b** Representative picrosirius red-stained LV collagen cross-sections from sham, vehicle and FPR agonist (Cmpd17b or Cmpd43, both 50 mg per kg per day i.p.) treated mice, 7 d post I-R. Scale bars: 500 µm (magnification x12.5, one representative mouse per group.

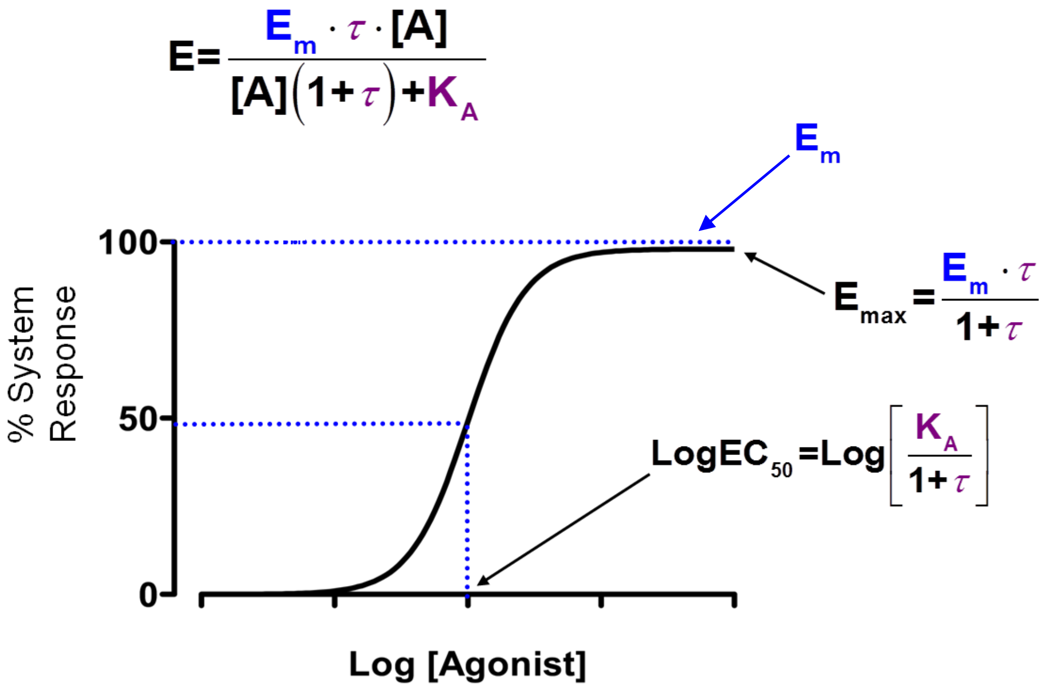

Supplementary Figure 8. To quantify signalling bias, agonist concentration–response curves were analysed by nonlinear regression using an operational model of agonism to define the Log(τ/K_A) "transduction coefficient" for each agonist for each pathway, where E_m is the maximal system response, K_A denotes the functional equilibrium dissociation constant of the agonist for the receptor, and τ the efficiency of coupling of the receptor its subsequent cellular stimulus-response transduction mechanism, as previously described.
